# The Highroads to Health
*"Hydropathy and Health; or, Sketches of Hydropathic Establishments and Health Resorts." By J. Ewing Ritchie ("Christopher Crayon"). London: Published for the Proprietor by W. Kent and Co., Paternoster Row. Price one shilling.


**Published:** 1889-02-09

**Authors:** 


### The Highroads to Health.*

Very few people can afford a *Wanderjahr;* an annual *Wandermonat* is the utmost we can get, and it is often a difficult question to people of moderate means where to spend it. Of course one can take lodgings at the seaside; but lodgings at the seaside—well, they are not ideal. The landlady has her "little ways," which are often uncomfortable and sometimes extortionate. The furniture also has its little ways. Drawers will not come out except after an amount of coaxing and coquetry that is little to the taste of a man who knows he is late for breakfast. The sofa once had springs—you know that, for their broken ends are barely concealed by the prickly horse-hair, and their fragmentary presence precludes all idea of repose. The chairs are untrustworthy about the legs, and everything of the nature of glass or china is so well cracked that it is sure to break while you are using it—and, of course, you must replace it, or pay considerably more than its value. Even if these disagreeables are not present in a marked degree, there is one characteristic of this once regular and traditional exodus—far be it from us to call it a disagreeable!—which prevents the annual holiday being the "thorough change" we all feel that we want. The whole family goes, perhaps to a place where they know no one, and the result is that the change does not give that most wholesome of all changes—change of society. The children are all right—they fraternise promiscuously on the sands; but papa and mamma can't do that; and the bigger boys and girls don't like to, however much interest Charlie feels in that girl with the violet eyes, or Edith in that young fellow who looks so handsome in his tennis things.

Besides, in transporting home to the seaside, home anxieties are included in the luggage. There are the usual duties, the ordering of the dinner, the looking after the children; home, though the dearest spot on earth, and so forth, is always a place where the duties outnumber the pleasures, even when it is home by the sea. So it comes about that sometimes the parents try to forget all the little cares and troubles, and all the years that have brought them, and go off together to resuscitate, not without success, the dear dead days of the honeymoon. Then the bachelor never did take his holiday with the rest of the family, and nowadays the spinster doesn't either. She wants to study the flora of the Highlands, or the geology of North Wales, and so takes her own way to holidays and health. And besides these, there are lonely folks of both sexes who have no families to go a-pleasuring with, and who have learnt by dreary experience that a holiday without companionship is no holiday at all. So they, and the other people we have spoken of, go off to a hydropathic establishment.

"Hydropathic" used to be a word of fear. It was held to signify a place where the natural liberty of the subject was interfered with, where the rash visitor was dosed with cold water, inside and out, till any disease he brought with him was washed out of him; and fancy pictured the establishments peopled by regiments of blue-nosed invalids, convalescent, but cold. Once, when hydropathy was first invented, or introduced, by Priessnitz—as told in "Christopher Crayon's" little book—there was a certain severity about it; there were washings as frequent as those of Naaman the Syrian; there was the compulsory swallowing of mineral waters—chalybeate, with the "flavour of warm flat irons," as Sam Weller described it; sulphurous, with odours suggesting subjection to an internal pillory; there was a Spartan simplicity of diet, worthy rather to be defined as dieting. But these things have passed away. A modern hydropathic is simply this: a splendid hotel, with every sanitary requirement carefully attended to, situated in splendid scenery, amid bracing air; where all rational amusements are provided; where the daily bath, dear to the British soul, is easily and luxuriously attainable; and where a liberal table manages to keep in consonance with the strictest laws of hygiene. As a rule, no intoxicants are to be had, and visitors are expected to keep what the Scotch call "elders' hours"—early to bed and early to rise, but not immoderately so at either end of the day. These restrictions may render hydropathics despicable in the eyes of a few, but for most of us they are additional attractions. A holiday spent in the best hotel of a lovely neighbourhood is considerably marred when, after a long excursion, our first sleep is disturbed by the parting words of a number of young fellows who have been "filling the flowing bowl until it doth run over."

Speaking of the Scotch, it is rather strange that hydropathy has taken such a firm hold on Caledonian soil. England and Wales support a fair number of hydropathics; but in proportion to its size, Scotland is much more thickly peopled with them. What is the strange attraction that draws the rather unsociable and not specially soap-loving Scottish soul to the gregarious hydropathics? Perhaps the Scotch are maligned, and deep down in their hearts they are all that they are supposed not to be, just as their stolid exteriors hide that passionate loyalty which makes so many of them Jacobites at the present day. However it be, nowhere does hydropathy succeed better than in Scotland; nowhere does sociability develop so fast; nowhere does acquaintance between young men and maidens, begun at a hydropathic, lead so frequently to the hymeneal altar.

But England, too, has its share of hydropathics and their amenities. Name any spot famous for the health-giving qualities of its air or water, and you will assuredly recall a large and handsome stone building, in appearance like a duke's mansion, standing in well-cared-for grounds—the hydropathic of the neighbourhood. If you have passed near enough you will have seen its numerous tennis-courts. If you had a friend staying there, you may chance to have received an invitation to a dance or a game at billiards in its recreation-rooms. For though hydropathics are built in districts marked by hygienic pretensions, their occupants are not generally invalids. They are the people who are a little worn out by the rush and turmoil of town life, but who recover in a few days, thanks to a pleasant and healthy mode of living, pleasant surroundings, and pleasant company, and devote the rest of their time to making merry. Each day has a character of its own. There are excursions to the hills and dales, the woods and waterfalls of the district; there are the before-mentioned dances in the evening, besides occasional outbursts of amateur theatricals and music—not such bad music for the sons and daughters of unmelodious John Bull. Often there is some energetic spirit among the guests who leads the amusements—an habitué of the place, who goes there season after season, till the hydropathic becomes his second home. Failing him, there is the manager, or the medical superintendent, or the matron, to set the ball a-rolling; at any rate, things never drag. Of course, those who choose may avoid these frivolities; they may interview the doctor every morning, and spend their days in one or other of the baths—Turkish, electric, medicated, or what not—with which the establishments are supplied; they may be resolutely ill, and determined to get their ailments' worth. But very few persist in such courses—except in the matter of interviewing the doctor, for we all like a chat with a courteous and intelligent gentleman. Gaiety is infectious, and a light heart has a sensible influence

* "Hydropathy and Health; or, Sketches of Hydropathic Establishments and Health Resorts." By J. Ewing Ritchie ("Christopher Crayon"). London: Published for the Proprietor by W. Kent and Co., Paternoster Row. Price one shilling.

on the liver. Hydropathics seem to have virtues as magical as the famous shrine at Kealaar:

"Those who came thither on crutches,
Now lightly dance on the ground;
And those now play on the viol
Who had not a finger sound."

Perhaps it is the air, perhaps it is the water, perhaps it is the treatment, perhaps it is the company; but for one reason or another hydropathics exercise a wholesome influence on all who frequent them, and those who go to them may reasonably congratulate themselves on having taken one of the high roads to health.

